# Rosmarinic Acid Protects Skin Keratinocytes from Particulate Matter 2.5-Induced Apoptosis

**DOI:** 10.7150/ijms.90814

**Published:** 2024-02-04

**Authors:** Herath Mudiyanselage Udari Lakmini Herath, Mei Jing Piao, Kyoung Ah Kang, Pincha Devage Sameera Madushan Fernando, Jin Won Hyun

**Affiliations:** Department of Biochemistry, College of Medicine and Jeju Research Center for Natural Medicine, Jeju National University, Jeju 63243, Republic of Korea.

**Keywords:** Rosmarinic acid, PM_2.5_, Oxidative stress, Apoptosis

## Abstract

**Background:** The exposure of the human skin to particulate matter 2.5 (PM_2.5_) results in adverse health outcomes, such as skin aging, wrinkle formation, pigment spots, and atopic dermatitis. It has previously been shown that rosmarinic acid (RA) can protect keratinocytes from ultraviolet B radiation by enhancing cellular antioxidant systems and reducing oxidative damage; however, its protective action against the adverse effects of PM_2.5_ on skin cells remains unclear. Therefore, in this study, we explored the mechanism underlying the protective effects of RA against PM_2.5_-mediated oxidative stress in HaCaT keratinocytes.

**Methods:** HaCaT keratinocytes were pretreated with RA and exposed to PM_2.5_. Thereafter, reactive oxygen species (ROS) production, protein carbonylation, lipid peroxidation, DNA damage, and cellular apoptosis were investigated using various methods, including confocal microscopy, western blot analysis, and flow cytometry.

**Results:** RA significantly inhibited PM_2.5_-induced lipid peroxidation, protein carbonylation, DNA damage, increases in intracellular Ca^2+^ level, and mitochondrial depolarization. It also significantly attenuated PM_2.5_-induced apoptosis by downregulating Bcl-2-associated X, cleaved caspase-9, and cleaved caspase-3 protein levels, while upregulating B-cell lymphoma 2 protein level. Further, our results indicated that PM_2.5_-induced apoptosis was associated with the activation of the mitogen-activated protein kinase (MAPK) signaling pathway and that MAPK inhibitors as well as RA exhibited protective effects against PM_2.5_-induced apoptosis.

**Conclusion:** RA protected HaCaT cells from PM_2.5_-induced apoptosis by lowering oxidative stress.

## Introduction

Globally, urban air pollution is a serious threat to public health. Fine particulate matter with aerodynamic diameter < 2.5 μm (PM_2.5_) is a constituent of airborne particulate matter that is primarily derived from industrial soot 1. The components of PM_2.5_ differ depending on its source, and PM_2.5_ emitted by diesel exhaust predominantly contains polycyclic aromatic hydrocarbons (PAHs), black carbon, and hydrocarbons (C_14_-C_35_) and their derivatives. PAHs in PM_2.5_ have a high mutagenic potential and can easily penetrate the skin via the appendageal route and the stratum corneum, thereby causing PM_2.5_-induced skin injury 2,3. Numerous studies have demonstrated that PM_2.5_ exposure increases the risk of cardiovascular and respiratory damage as well as neurotoxicity 4,5. Further, PM_2.5_ has been linked to various skin disorders, including acne, atopic dermatitis, and skin aging 6,7. It has also been shown that exposure to PM_2.5_ induces reactive oxygen species (ROS) generation in keratinocytes, and this excessive intracellular ROS generation induces cell damage owing to oxidative stress 3,8, which can considerably harm nucleic acids, proteins, lipids, cell membranes, and organelles, such as the mitochondria, and even induce apoptosis 9.

Most phenolic compounds can function as antioxidants or free-radical scavengers. Specifically, rosmarinic acid (RA), a naturally occurring hydroxylated polyphenolic compound that is commonly found in* Rosmarinus officinalis,* exhibits antimicrobial, anti-inflammatory, antioxidative, antiapoptotic, and antitumor activities 10-12. Our previous study demonstrated that it exerts cytoprotective effects against ultraviolet B radiation by modulating cellular antioxidant systems in keratinocytes and ameliorating oxidative damage 11,12. However, the cytoprotective action of RA against PM_2.5_ remains unclear. Therefore, in this study, we aimed to explore the cytoprotective effect of RA on PM_2.5_-induced skin damage.

## Materials and Methods

### Reagents

RA (100% purity), primary antibodies for Bax, Bcl-2, PARP, ERK, phospho-ERK, and p38 were procured from Santa Cruz Biotechnology (Dallas, TX, USA); diesel particulate matter NIST 1650b (PM_2.5_), NAC (antioxidant), Hoechst 33342, Z-VAD-FMK (caspase inhibitor), SB203580 (p38 inhibitor), trypan blue solution and avidin-TRITC were procured from Sigma-Aldrich Inc. (St. Louis, MO, USA). MTT and DMSO were purchased from Amresco LLC (Solon, OH, USA). H_2_DCFDA, Fluo-4 AM and DPPP were purchased from Molecular Probes (Eugene, OR, USA). JC-1 was purchased from Invitrogen (Carlsbad, CA, USA). SP600125 (JNK inhibitor) and U0126 (MEK inhibitor) were obtained from Tocris (Bristol, UK) and Calbiochem (La Jolla, CA, USA), respectively. Actin, caspase-3, caspase-9, phospho-p38, JNK and phospho-JNK primary antibodies were purchased from Cell Signaling Technology (Beverly, MA, USA).

### Preparation of RA and PM_2.5_

Stock solutions of RA and PM_2.5_ were prepared using DMSO. Specifically, the prepared PM_2.5_ stock solution (25 mg/mL) was subjected to sonication for 30 min to prevent the particles from clustering 13.

### Cell culture

The human skin HaCaT cell line was procured from Cell Lines Service (Eppelheim, Germany). The cells were cultured according to standard procedures at 37 °C in an incubator with 5% CO_2_ and full humidity. Further, the culture medium, DMEM, was supplemented with 10% heat-inactivated FBS and an antibiotic-antimycotic mix 14.

### MTT assay

The cells were incubated with MAPK pathway-targeted chemical inhibitors (50 nM U0126; 10 µM SB203580; and 5 µM SP600125) with or without 2.5 μM RA for 30 min and then exposed to 50 μg/mL PM_2.5_ for 24 h at 37 °C. Thereafter, MTT solution was added to each well after which incubation was further performed at 37 °C for 4 h. The purple precipitate thus obtained was dissolved by adding DMSO and cellular metabolic activity measurements were performed at 540 nm using a VersaMax ELISA microplate reader (Molecular Devices, Sunnyvale, CA, USA) 14.

### Trypan blue assay

Cells were exposed to 30 μM Z-VAD-FMK with or without 2.5 μM RA for 30 min and then exposed to PM_2.5_ at 50 μg/mL for 24 h. After this treatment, the cells were stained using 0.1% trypan blue solution and observed using a fluorescence microscope 15.

### ROS measurement

Cells were treated with 2.5 μM RA or 1 mM NAC for 30 min and then exposed to 50 μg/mL PM_2.5_ for 24 h. Then, for total ROS and superoxide anion detection, 25 μM H_2_DCFDA and 10 μM DHE, respectively, were added to the culture medium and fluorescent cells were analyzed using a flow cytometer (Becton Dickinson, Mountain View, CA, USA) 3,14. Further, cellular H_2_O_2_ level was measured via the ROS-Glo™ H_2_O_2_ assay (Promega, Madison, WI, USA) according to the manufacturer's protocols 16.

### Lipid peroxidation

Cells were stained with the fluorescent probe DPPP (5 μM), and stained cells were observed under a confocal laser scanning microscope (Carl Zeiss, Oberkochen, Germany) 8.

### Protein carbonylation

Protein carbonylation level was assessed using the OxiSelect™ protein carbonyl ELISA kit (Cell Biolabs, San Diego, CA, USA) following the guidelines provided by the manufacturer 8.

### Comet assay

Cells were collected on a slide, electrophoresed (25 V, 300 mA), stained with ethidium bromide, and observed under a fluorescence microscope equipped with image analysis software (Kinetic Imaging, Komet 5.5, UK). The percentage of total fluorescence in the comet tail and the length of the tail were measured in 50 cells/slide 17.

### 8-Oxoguanine (8-oxoG)

The avidin-TRITC conjugate was used to detect 8-oxoG, a biomarker of oxidative stress-induced DNA damage. Stained cells were observed using a confocal microscope 8.

### Intracellular Ca^2+^ level

To detect intracellular Ca^2+^ level, cells were stained with 10 μM Fluo-4 AM and fluorescence intensity measurements were performed via flow cytometry and confocal microscopy 3.

### Mitochondrial membrane potential (Δψ_m_)

The cells were first labeled with JC-1, a lipophilic cationic fluorescent dye 3. Thereafter, mitochondrial membrane potential (Δψ_m_) was measured via confocal microscopy.

### Western blot analysis

Extracted cell lysates were electrophoresed on SDS-polyacrylamide gel to separate proteins. Thereafter, the separated proteins were electroblotted to nitrocellulose membranes, which were then incubated with the appropriate primary antibodies (1:1,000), followed by incubation with a secondary antibody. Protein expression levels were then detected using an enhanced chemiluminescence plus western blot detection system (GE Healthcare Life Sciences, Buckinghamshire, UK) 14.

### Hoechst 33342 staining

Cells were stained with a DNA-specific fluorescent dye, Hoechst 33342 and examined using a fluorescence microscope (Media Cybernetics, Rockville, MD, USA). Finally, the proportion of apoptotic cells was quantified 14.

### Annexin V/PI staining assay

Apoptotic cells were quantified via flow cytometry using the Alexa Fluor^TM^ 488 annexin V/dead cell apoptosis kit (Invitrogen, Thermo Fisher Scientific, Inc.) in accordance with the guidelines provided by the manufacturer 18.

### Statistical analysis

Statistical analyses were performed using sigmaStat version 3.5 (Systat SoftwareInc., San Jose, CA, USA). *p* < 0.05 indicated significance.

## Results

### RA protected skin cells by lowering PM_2.5_-induced increases in ROS levels

**Figure 1:**
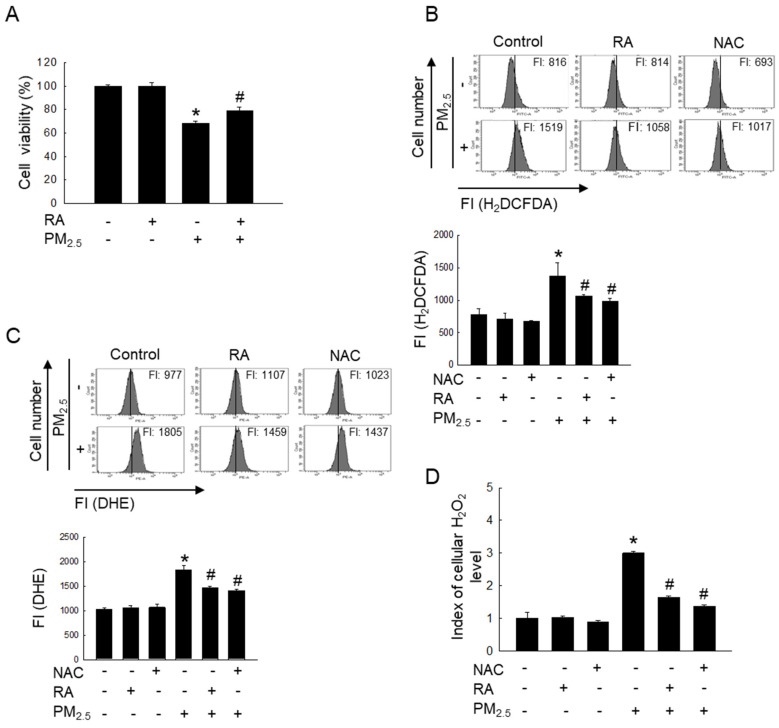
RA ameliorated PM_2.5_-mediated cell death and ROS generation. (A) Cell viability measured via the MTT assay. (B) Cellular ROS amount detected via H_2_DCFDA staining. (C) Superoxide anion detected via DHE staining. (D) Cellular H_2_O_2_ level measured via the ROS-Glo™ H_2_O_2_ assay. (B, C) FI means fluorescence intensity. (A-D) *p < 0.05 vs. control; ^#^*p* < 0.05 vs. PM_2.5_.

Reportedly, RA at 2.5 μM does not induce any form of cytotoxicity in HaCaT cells; it maintains cell viability at approximately > 95% 11. Therefore, 2.5 μM RA was used as the optimal RA concentration in this study. The exposure of HaCaT cells to PM_2.5_ significantly reduced cell viability; however, treatment with RA significantly reversed the PM_2.5_-induced loss of cell viability (Figure 1A). Further, RA pretreatment significantly lowered the PM_2.5_-induced increase in the levels of ROS, including superoxide anion and hydrogen peroxide (Figures 1B-D).

### RA attenuated PM_2.5_-induced macromolecular damage

**Figure 2:**
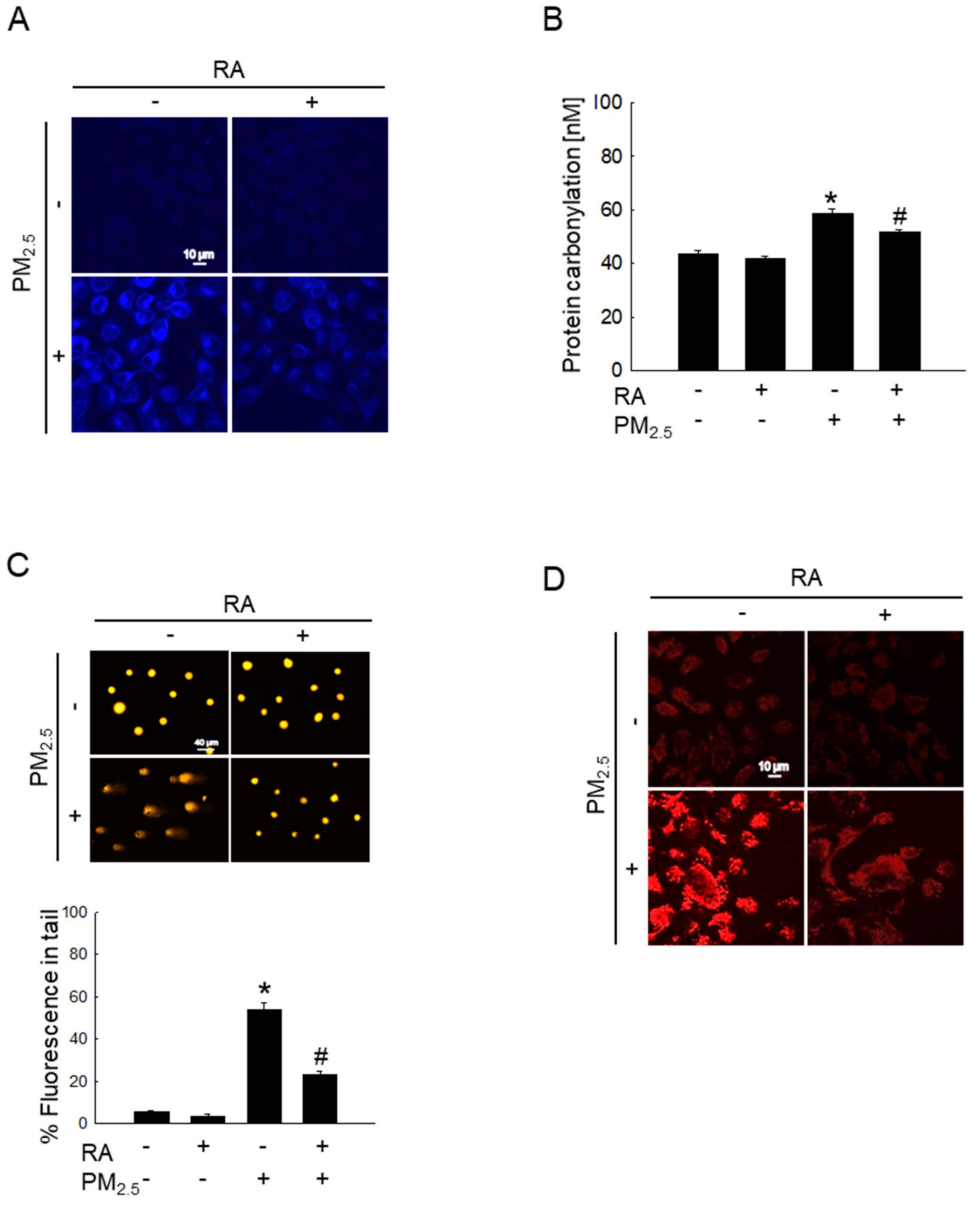
RA prevented oxidative damage to intracellular molecules caused by PM_2.5_. (A) DPPP staining for the detection of lipid oxidation performed. (B) Protein carbonylation measured using the protein carbonyl ELISA kit. (C, D) DNA damage observed via (C) a comet assay and (D) avidin-TRITC staining. (B, C) *p < 0.05 vs. control; ^#^*p* < 0.05 vs. PM_2.5_.

Oxidative stress significantly increases intracellular macromolecular damage 19. In this study, we examined the protective properties of RA against macromolecular damage caused by PM_2.5_. The examination of PM_2.5_-induced lipid peroxidation via DPPP staining showed the significant enhancement of fluorescence intensity in the PM_2.5_-treated group; however, pretreatment with RA significantly decreased this fluorescence intensity (Figure 2A). Further, PM_2.5_-mediated oxidative stress resulted in a significant increase in protein carbonylation level, while RA pretreatment prevented this phenomenon (Figure 2B). Additionally, the examination of PM_2.5_-induced DNA damage via the comet assay showed that PM_2.5_-induced oxidative stress increased comet tail length and tail fluorescence percentage; however, these observations were reversed following RA pretreatment (Figure 2C). 8-Oxoguanine, a major form of oxidative DNA damage, was examined using an avidin-conjugated TRITC reagent 20. The confocal microscopy images obtained thereafter revealed severe DNA lesions in the PM_2.5_-exposed cells; however, pretreatment with RA ameliorated this effect (Figure 2D).

### RA decreased PM_2.5_-induced apoptotic cell death

**Figure 3:**
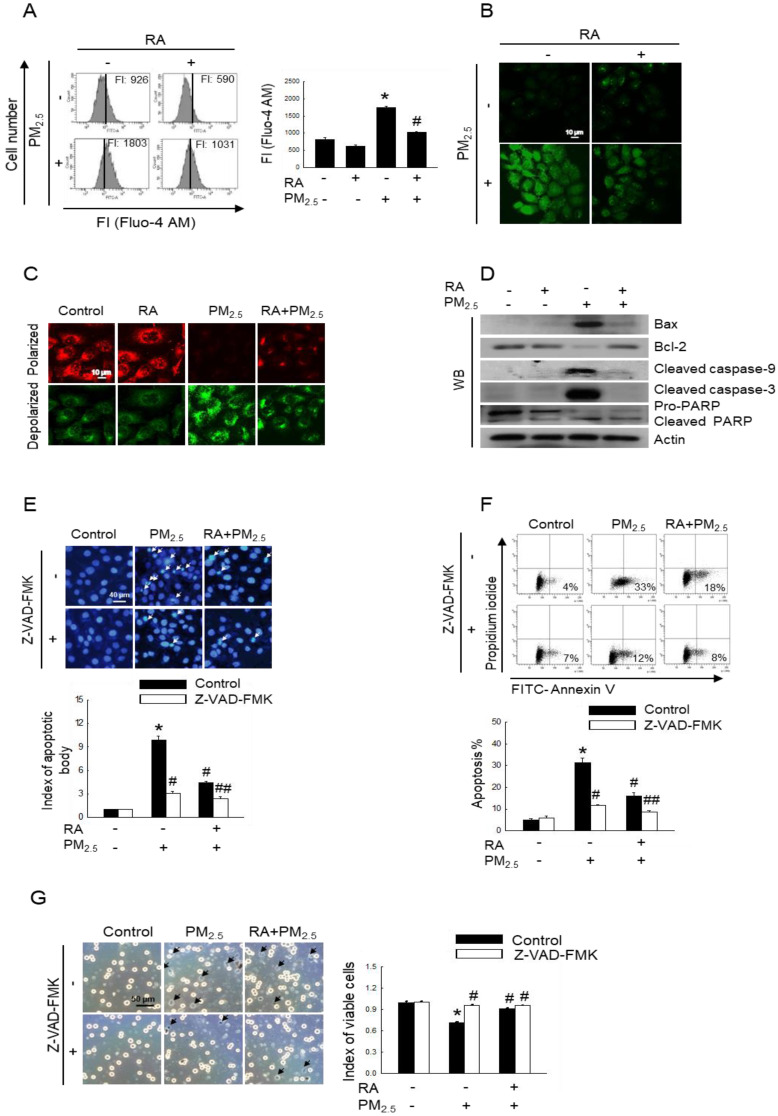
RA reduced apoptosis triggered by PM_2.5_. (A, B) Ca^2+^ level after Fluo-4 AM staining detected via (A) flow cytometry and (B) confocal microscopy. (C) Δψ_m_ following JC-1 staining detected using a confocal microscope. (D) Bax, Bcl-2, cleaved caspase-9, cleaved caspase-3, and PARP protein expression levels detected via western blot analysis. Actin used as the loading control. (E, F) Apoptotic levels observed via (E) Hoechst 33342 staining and (F) annexin V/PI staining. Apoptotic bodies indicated using arrows. (G) Cell viability detected using trypan blue staining. (A) FI means fluorescence intensity. (A, E-G) *p < 0.05 vs. control; ^#^*p* < 0.05 vs. PM_2.5_; ^##^* p* < 0.05 vs. RA+PM_2.5_.

Oxidative stress results in increased cytoplasmic Ca^2+^ concentration, and such elevated Ca^2+^ level can damage mitochondria and nuclei, leading to apoptosis 21. After labelling cells with Fluo-4 AM, we measured intracellular Ca^2+^ level via flow cytometry and confocal imaging. Thus, we observed an increased fluorescence intensity for cells exposed to PM_2.5_, indicating increased Ca^2+^ level; however, pretreatment with RA resulted in a decrease in Ca^2+^ level, as depicted in Figure 3A and B. Mitochondria act as apoptosis-regulating centers. PM_2.5_-induced excessive ROS generation can induce mitochondrial oxidative damage, and reportedly, severe mitochondrial damage is associated with mitochondrial malfunction and apoptosis 22. Our results showed that PM_2.5_ enhanced mitochondrial depolarization, which was reversed by pretreatment with RA (Figure 3C). Previous studies have shown that PM_2.5_ enhances keratinocyte apoptosis by activating the caspase signaling pathway 15,23. As shown in Figure 3D, PM_2.5_ exposure upregulated Bax, cleaved caspase-9, cleaved caspase-3, and cleaved PARP protein levels, while pretreatment with RA downregulated these proteins. In contrast, the expression level of antiapoptotic protein, Bcl-2 was decreased following PM_2.5_ exposure; however, it was enhanced owing to RA pretreatment. To verify the effect of caspase activation on apoptosis, HaCaT cells were pretreated with a caspase inhibitor (Z-VAD-FMK) with or without RA, both of which are associated with decreases in the amounts of apoptotic bodies (Figure 3E). As was observed for Hoechst 33342-staining, the percentage of annexin V-stained cells in the PM_2.5_ group increased and this effect was ameliorated by Z-VAD-FMK or/with RA pretreatment (Figure 3F). Trypan blue staining further revealed that Z-VAD-FMK and RA pretreatment ameliorated PM_2.5_-induced decreases in cell viability (Figure 3G).

### RA downregulated the PM_2.5_-activated MAPK signaling pathway

**Figure 4:**
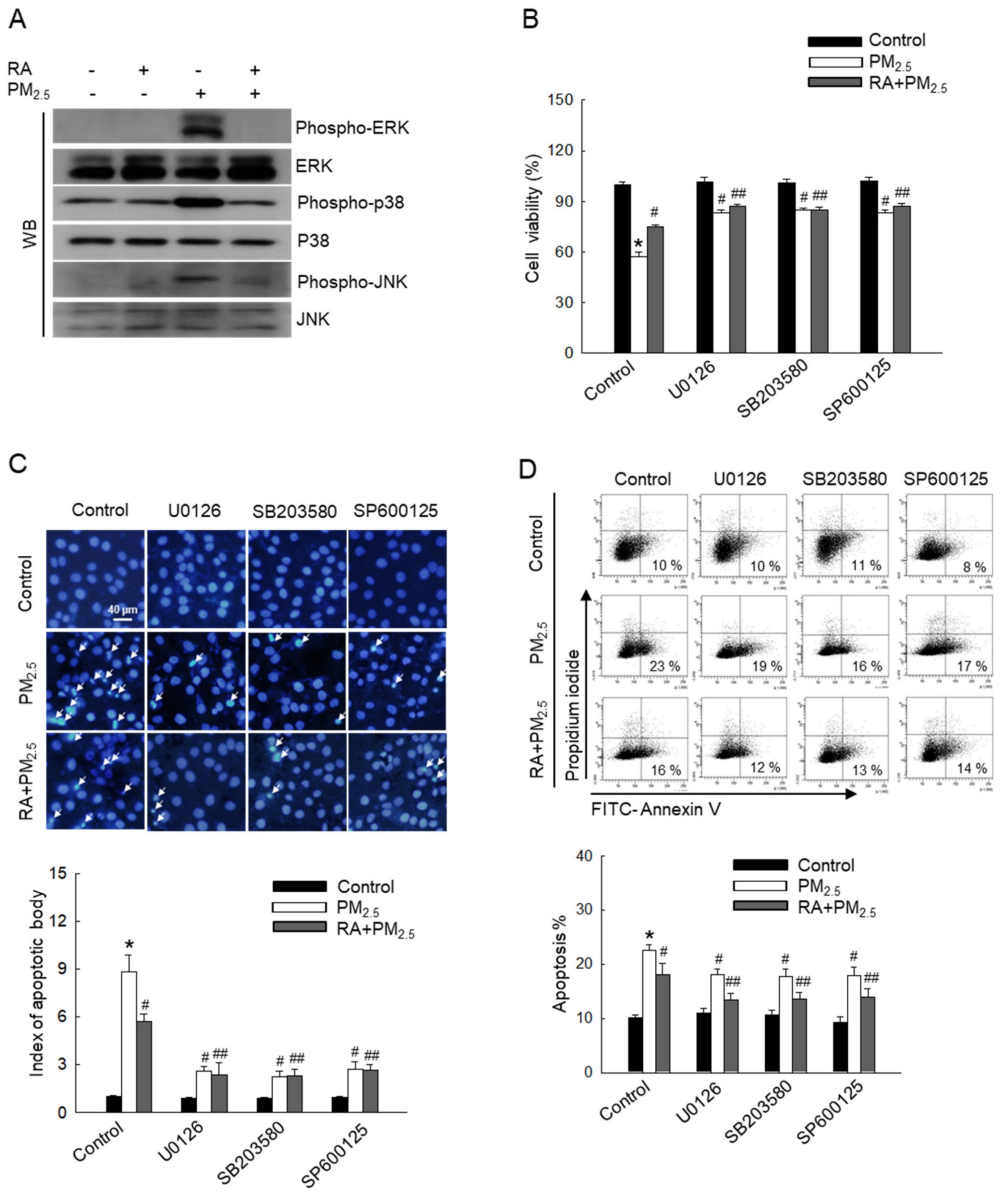
RA lowered PM_2.5_-induced activation of the MAPK signaling pathway. (A) Western blot analysis performed for detection of ERK, p38, and JNK protein expression levels. Total ERK, p38 and JNK protein levels used as the loading control. (B) Cell viability detected via the MTT assay. Apoptotic levels observed via (C) Hoechst 33342 staining and (D) Annexin V/PI staining. Apoptosis bodies indicated using arrows. (B-D) *p < 0.05 vs. control; ^#^*p* < 0.05 vs. PM_2.5_; ^##^* p* < 0.05 vs. RA + PM_2.5_.

MAPK signaling pathways regulate various biological processes, such as apoptosis 24. To further investigate the effect of PM_2.5_ on MAPK signaling pathway-mediated apoptosis, MAPK signaling cascade-associated proteins, ERK, p38, and JNK were detected via western blot analysis (Figure 4A). Thus, we observed that PM_2.5_ elevated the levels of phospho-ERK, phospho-p38, and phospho-JNK in comparison with the control treatment. However, RA pretreatment reversed this effect. Cells pretreated with each ERK, p38, and JNK inhibitor (U0126, SB203580, and SP600125) exhibited decreased PM_2.5_-induced cytotoxicity and apoptosis, similar to cells pretreated with RA (Figure 4B-D). These results demonstrated that RA ameliorates PM_2.5_-induced apoptosis by downregulating the PM_2.5_-induced MAPK signaling pathway.

## Discussion

Skin keratinocytes constitute the primary barrier against environmental stressors and exposure of keratinocytes to PM_2.5_ could lead to increased ROS production 25. Recent studies have demonstrated that PM_2.5_ may penetrate the skin and directly affect viable skin cells, including keratinocytes 9,25. Therefore, in this study, we aimed to explore the protective effects of RA against PM_2.5_-induced damage in HaCaT cells. Our previous study showed that at concentrations up to 2.5 μM, RA does not exert any cytotoxic effects on HaCaT and that at 2.5 μM, RA scavenges up to 60% of intracellular ROS 11. Thus, in this study, we used 2.5 μM as the optimal RA concentration. Further, based on our previous study, which showed that the optimal PM_2.5_ exposure concentration required to induce excessive ROS generation in HaCaT cells is 50 μg/mL 10. In this study, we observed that RA significantly reversed PM_2.5_-induced decreases in the viability of HaCaT cells. RA also significantly reduced PM_2.5_-induced increases in ROS levels.

PM_2.5_-induced excessive ROS generation can trigger oxidative macromolecular damage, leading to apoptosis 26. In the lipid peroxidation process, oxidants, such as free radicals or ROS attack several carbon-carbon double bonds in polyunsaturated fatty acids, initiating the removal of hydrogen from carbon to produce water and fatty acid radicals. The unstable fatty acid radicals thus obtained react with molecular oxygen to form lipid peroxyl radicals and hydroperoxides 27. This membrane lipid peroxidation alters the physical properties of lipid bilayers, and consequently, affects membrane permeability, lipid-lipid interactions, ion gradients, and membrane fluidity 28. Thus, lipid peroxidation negatively affects cellular functions. Additionally, oxidative stress caused by ROS overproduction results in protein carbonylation owing to protein oxidation or DNA modification 8. In this study, we observed that RA exhibited a protective effect against PM_2.5_-induced macromolecular damage by diminishing ROS.

Owing to oxidative stress, Ca^2+^ flows into the cytoplasm from internal cellular stores, such as the sarcoplasmic reticulum/endoplasmic reticulum and can also be imported from extracellular spaces. As the Ca^2+^ concentration in the cytoplasm increases, Ca^2+^ flows into the mitochondria and nuclei. The accumulation of Ca^2+^ in mitochondria and nuclei as such disrupts normal cell metabolism, leading to apoptosis 21,29. In this study, we observed that RA significantly attenuated oxidative stress-mediated cellular Ca^2+^ influx. Mitochondria play a major role in the onset of apoptosis. Moreover, PM_2.5_-induced oxidative stress leads to mitochondrial damage, including changes in Δψ_m_ 15,19,30. In this study, we observed that RA regulated Δψ_m_ and facilitated the normal functioning of mitochondria. It has been shown that mitochondrial damage is associated with Bcl-2, which maintains the integrity of the mitochondrial membrane. The loss of Δψ_m_ induces the release of apoptotic factors into the cytosol. Further, the balance between the pro-apoptotic protein, Bax and the antiapoptotic proteins of the Bcl-2 family serves as the determining factor for the activation or inhibition of the caspase cascade. Specifically, caspase-3 initiates the intrinsic apoptotic pathway and cleaves nuclear proteins, such as PARP 31,32. It has also been shown that PM_2.5_ upregulates apoptotic protein levels while downregulating the expression of antiapoptotic proteins 8,15. In this study, we observed that RA reversed these effects.

The MAPK signaling cascade plays a crucial role in mediating cellular stress response. Previous reports have shown that PM_2.5_ activates MAPK pathway-associated proteins via ROS-mediated pathways 15,19. The activation of p38 and JNK in response to PM_2.5_ is thought to induce apoptosis 33. In contrast, the ERK pathway plays a dual role in cell survival and death. Further, recent studies have shown that continuous ERK activation can promote apoptosis 15,34. Further exploration in this regard using ERK, p38, and JNK inhibitors revealed that RA pretreatment reduced the phosphorylation of ERK, p38, and JNK, similar to their respective inhibitors, leading to a decrease in the levels of apoptotic bodies.

## Conclusion

**Figure 5:**
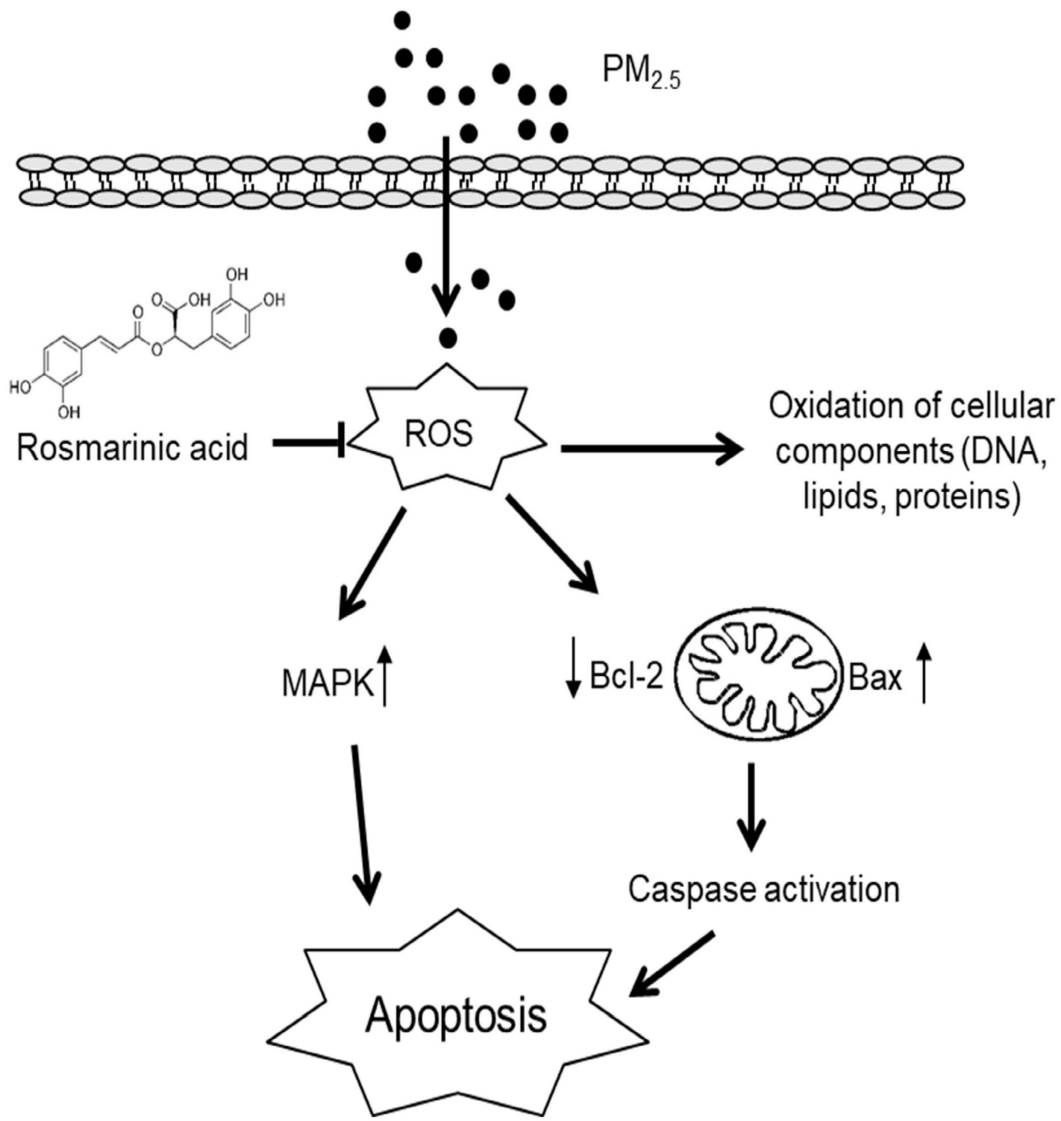
Summary of the protective effects of RA against PM_2.5_-induced skin cell damage. RA ameliorated PM_2.5_-induced intracellular ROS levels, hence attenuated oxidative damage to DNA, lipids, and proteins. Further, RA downregulated mitochondria-mediated caspase activation and the MAPK signaling pathway, and thus ameliorated apoptosis.

In this study, we investigated the effects of RA on PM_2.5_-induced damage in keratinocytes. Our results revealed that PM_2.5_ exacerbated skin cell damage by increasing ROS generation and activating apoptotic pathways; however, RA ameliorated these observed PM_2.5_-induced effects. Thus, it showed protective effects on skin cells against PM_2.5_-induced damage (Figure 5).

## Abbreviations

PM_2.5_: particulate matter 2.5
RA: rosmarinic acid
ROS: reactive oxygen species
PAHs: polycyclic aromatic hydrocarbons
Bax: Bcl-2-associated X protein
Bcl-2: B-cell lymphoma 2
ERK: extracellular signal-regulated kinase
PARP: poly-ADP ribose polymerase
MAPK: mitogen-activated protein kinase
avidin-TRITC: avidin-tetramethylrhodamine isothiocyanate
MTT: thiazolyl blue tetrazolium bromide
DMSO: dimethyl sulfoxide
H_2_DCFDA: 2′,7′-dichlorodihydrofluorescein diacetate
DHE: dihydroethidium
DPPP: diphenyl-1-pyrenylphosphine
PI: propidium iodide
JC-1: 5,5′,6,6′-tetrachloro-1,1′,3,3′-tetraethylbenzimidazolylcarbocyanine iodide
NAC: N-acetyl cysteine
JNK: c-Jun *N*-terminal kinase
DMEM: Dulbecco-modified Eagle medium
Fluo-4 AM: fluo-4-acetoxymethyl ester
MEK: mitogen-activated protein kinase kinase
FI: fluorescence intensity
